# Domestic violence among married women of reproductive age in Zimbabwe: a cross sectional study

**DOI:** 10.1186/s12889-020-8447-9

**Published:** 2020-03-18

**Authors:** Joseph Lasong, Yuan Zhang, Kahindo P. Muyayalo, Olivia Adhiambo Njiri, Simon Afewerki Gebremedhin, Chrissie S. Abaidoo, Chun Yan Liu, Huiping Zhang, Kai Zhao

**Affiliations:** 1 Institute of Reproductive Health/Center for Reproductive Medicine, Tongji Medical College, Huazhong University of Science and Technology, Wuhan, China; 2 Department of Parasitology, Tongji Medical College, Huazhong University of Science and Technology, Wuhan, China; 3 Department of Public Health, Tongji Medical College, Huazhong University of Science and Technology, Wuhan, China; 4 School of Medical Sciences, Kwame Nkrumah University of Science and Technology, Kumasi, Ghana

**Keywords:** Domestic violence, Public health trends, Zimbabwe, Risk factors, Africa

## Abstract

**Background:**

Domestic violence does not only violate women’s fundamental human rights but it also undermines them from achieving their fullest potential around the world. This study was conducted to assess trends and factors associated with domestic violence among married women of reproductive age in Zimbabwe.

**Method:**

This was a cross-sectional study which used secondary data obtained from 2005/06, 2010/11 and 2015 Zimbabwe Demographic and Health Surveys (ZDHS). Respondents ranged from married or living with a partner (15–49 years). Multiple logistic regression analysis was used to examine factors associated with domestic violence.

**Results:**

Out of 4472 women who were currently married, 1907 (42.7%) had ever experienced one form of domestic violence (physical, emotional and sexual violence). Women aged 40–49 was deemed a protective factor against domestic violence. Risk of domestic violence was higher among working women than unemployed women [AOR = 1.35; *p* ≤ 0.047]. Women who drink alcohol significantly risk experiencing domestic violence compared to their non-drinking counterpart; also women whose husbands drink alcohol were at higher risk of experiencing domestic violence [AOR = 1.35; *p* ≤ 0.001]. Domestic violence was higher among women whose husbands have ever experienced their fathers beating their mothers and significant for women whose husbands have more than one wife (polygamy) [AOR = 1.35; *p* ≤ 0.001]. High parity (5 or more children) was also a risk factor for domestic violence among the studied population [AOR = 1.35; *p* ≤ 0.038].

**Conclusion:**

Domestic violence was found to be strongly associated with women whose husbands drink alcohol, products of abusive parents/father beating their mother and/or polygamous marriage (had more than one wife). Domestic violence still remains a challenge and a more biting policy efforts are needed to eradicate this public health canker in Zimbabwe.

## Background

Women have rights just like any other human being; they have right to live, right not to be subjected to torture or inhumane or degrading treatment or punishment, right to equal protection, right to liberty and personal security, right to equal protection under the law, right to equality in the family, right to the highest standard attainable of physical, mental health and right to justice [1]. Violence against women violates these rights and fundamental freedoms of women and promotes gender inequality in society [1]. Globally, Sub-Saharan African countries have documented violence against women [2]. Domestic violence is predominant in most of sub-Saharan Africa, with a total prevalence of 36% above the global average of 30% [3]. Majority of women in Africa are prone to lifetime partner violence (45.6%) and sexual abuse (11.9%) than elsewhere [3]. Zimbabwe enacted the domestic violence Act (Chapter 5:16) on 26th February, 2007. In the Zimbabwean setting, domestic violence has been defined as any act or omission or commission or behavior of a respondent in case it harms or injures or endangers the health, safety, life or well-being, whether mental or physical, of the aggrieved person or tends to do so and includes causing physical abuse, sexual abuse, verbal and emotional abuse [4].

In 1993, the United Nations declared total elimination of domestic violence with member countries as key signatories to end violence against women globally. Consequently, 119 countries have passed their own domestic violence laws [5]. Despite the enforcement of this convention and country-level laws, cases of domestic violence is globally on the ascendancy. An estimated 1.3 million people (especially women) die annually as a direct result of domestic violence; accounting for 2.5% of global mortality [6]. Women accounts for 80% of domestic violence victims regardless of income, age or education with intimate partner violence accounting for the majority of women’s experiences of violence [7].

Zimbabwe has limited published empirical evidences on domestic violence. Trends and factors associated with domestic violence are often established as culture-specific and geographically diverse; hence the need for this study to ascertain potential cultural dynamics and geographical diversity as regards domestic violence in the African nation. Consistently, literature is replete with differentially associated socio-cultural and geo-traditional factors with domestic violence across the world. Several studies have reported that women are less educated, and pregnant with husbands who drink alcohol have higher risks to domestic violence [8–16]. Women who are less empowered, resides in rural areas and married to husbands who are from abusive homes, thus, ever experienced their father beating their mothers stands higher risks of domestic violence [10, 15, 17–24]. Conversely, several literature evidenced that higher educational level for both women and men (husbands), women from wealthy families, empowered women and residing in urban areas were protective factors against domestic violence [11–13, 15, 19, 21, 25, 26].

Furthermore, domestic violence was less likely in women whose husbands had secondary education [7]. Another study conducted by Abramsky et al. [27] found that young age, attitudes supportive of wife beating, having outside sexual partners, experiencing childhood abuse, growing up with domestic violence, and experiencing or perpetrating other forms of violence in adulthood and co-habitation were risk factors of domestic violence while secondary education and formal marriage were protective factors against domestic violence [27].

Domestic violence does not only have an impact on the life of the victim but it also affects the economy of the country which includes costs in relation to police, hospital and health services, legal costs, and social support services [7]. The aim of this study was to explore the trends and factors associated with domestic violence among married women in Zimbabwe using a national representative data from DHS. Addressing specific factors associated with domestic violence will provide evidence-based information to influence policy to prioritize a more robust and holistic interventions and also provide evidence for future development of public health policy recommendations against domestic violence.

## Methods

### Data source and methods

This was secondary data sourced from 2005 to 2006, 2010–2011 and 2015 Zimbabwe demographic and health survey (ZDHS). The survey was designed to provide data for monitoring the population and health situation in Zimbabwe. The survey gives national representative samples of women aged 15–49 years selected at a household and were interviewed. The response rate of 2005–2006, 2010–2011 and 2015 ZDHS ranged from 93 to 96%.

The recent data (2015 ZDHS) was used to identify factors associated with domestic violence among married or co-habitating partners (non-pregnant women of reproductive age; 15–49 years). A sample size of 9, 955 women of reproductive age (15–49) were restricted to 10,534 households and were interviewed. This study was limited to married or co-habitating (non-pregnant women), after excluding pregnant, not married women and missing data; 4472 samples were included for analyses.

### Study variables

#### Dependent variable

The outcome variable was domestic violence and binary in nature comprising those who have experienced and never experienced domestic violence (0 = No, 1 = Yes). It measured whether married women have ever experienced domestic violence or not. Physical violence plus emotional violence plus sexual violence constituted Domestic Violence.

The survey asked the following questions which were used to create the physical violence variable by the husband or partner (7 questions were asked) **1.** Ever been slapped?; **2.** Ever been twisted in your arm or pulled your hair?*;***3.** Ever been pushed, shook, or threw something at you?*;***4.** Ever been punched with his fist or with something that could hurt you?*;***5.** Ever been kicked, dragged or beaten up?*;***6.** Ever been tried to choke you or burn you on purpose?*;***7.** Ever been threatened or attacked with a knife, gun, or any other weapon? *Physical violence was indicated* if a woman scored from 1 to 7 and was coded as “1” and physical violence was not present if a woman scores “0” and it was coded as “0”. *Sexual violence (variable by husband or partner) was created from the following questions;***1.** Ever been physically forced into unwanted sex? **2.** Ever been forced into other unwanted sexual act? *Sexual violence was indicated if a woman scores* “1” or “2” and it was coded as “1”. Scores of “0” indicated no sexual violence and it was coded as “0”. Emotional violence variable (by husband or partner) was formulated from the following questions; **1.***Ever been humiliated?;***2.***Ever been threatened with harm?;***3***. Ever been insulted or made to feel bad?.* Score of “1” to “3” indicated emotional violence and it was coded as 1 and score of “0” showed no emotional violence and it was coded as “0”.

#### Independent variables

Several independent variables were used to predict domestic violence among married women in Zimbabwe. The variables were age (15–19, 20–29, 30–39, and 40–49 years), residence (rural/ urban), respondent (husband/partner) educational level (no education, primary, secondary and higher education), wealth index (poor, middle and rich), religion (no religion, Christians, Muslims, Traditional and others), smoking (Yes/No), respondent currently working (Yes/No), respondent drinking alcohol (Yes/No), partners occupation (not working, agriculture and non-agriculture), father beat mother (Yes/No), women empowerment (Yes/No), parity (0, 1–2, 3–4 and 5+), media exposure (Yes/No), partner drinking alcohol (Yes/No), age difference; which was calculated by subtracting the husband/ partner age with respondent age, and grouped as (woman same age or older than man, man older < 10 years and man older than > = 10 years) and number of wives (polygamous/ monogamous) .

Mass media exposure variable was formed from the following questions: **1.***How frequent do you listen to radio?;***2.***Do you watch television?;***3***. How frequent do you read newspapers or magazine?;* A score of “0” showed not exposed and was coded as “0”, a score of 1 indicated exposed at least one media and was codes as “1”, score of “2” measured exposure at least to two media and it was coded as “2” and score of “3” measured exposure to all media and it was coded as “3”. Women empowerment variable was created from the following questions: **1.***Who decides on respondent health care?;***2.***Who decides on large household purchase?;***3.***Who decides on household purchase for daily needs?;***4.***Who decides on visiting family or relatives?.* A score from 1to 4 indicated women empowerment and it was coded as “1” and a score of “0” indicated no women empowerment and it was coded as “0”.

#### Statistical analyses

The International Business Machine Statistical Package for Social Scientist (IBM-SPSS) software (ver. 22) was used for data analyses. Descriptive statistics were performed using cross-tabulation to show frequency distribution of variables and categorical variables were displayed as percentages. Univariate analysis was performed to examine the association between dependent variable (domestic violence) and each independent variable. Subsequently, multiple logistic regression analysis was performed and factors associated with domestic violence were identified after controlling for potential confounding factors. Results were presented as Odds ratio (OR), 95% confidence interval (CI) and *p*-value was used to measure the statistical significance (*p* ≤ 0.05).

## Result

### Socio-demographic characteristics

Table 1 shows the socio-demographic characteristics of 4472 Zimbabwean women of reproductive age 15–49 years; 42.7% (1909) had ever experienced domestic violence (physical, sexual or emotional violence). The average age of the respondents was 31.5 ± 7.9 years. Above three fourth, 76.8% (3436) of the respondents were within the younger age group of 20–39 years. About six in ten, 59.0% (2640) of the women were living in the rural areas. A significant number of the women, 63.5% (2840) and their partners, 65.9% (2947) had secondary education and above half, 50.4% (2252) may be described as within the rich wealth index group. Nearly all 93.5% (4183) of the respondents were Christians; a paltry number of the women, 0.4% (19) reported smoking cigarettes, and drink alcohol, 10.4% (466).

**Table 1:** Socio-demographic characteristics and association with Domestic Violence among women of reproductive age in Zimbabwe (2015)

Socio-demographic variable	Total	No	Yes	χ^**2**^	***p***-Value
	4472	2563 (57.3)	1909 (42.7)		
	N (%) **	N (%)*	N (%) **		
**Age**				28.37	< 0.001
15–19	209 (4.7)	114 (54.5)	95 (45.5)		
20–29	1719 (38.4)	932 (54.2)	787 (45.8)		
30–39	1717 (38.4)	978 (57.0)	739 (43.0)		
40–49	827 (18.5)	539 (65.2)	288 (34.8)		
**Residence**				0.004	0.949
Urban	1832 (41.0)	1051 (57.4)	781 (42.6)		
Rural	2640 (59.0)	1512 (57.3)	1128 (42.7)		
**Highest education level**				26.36	< 0.001
No Education	54 (1.2)	31 (57.4)	23 (42.6)		
Primary	1245 (27.8)	690 (55.4)	555 (44.6)		
Secondary	2840 (63.5)	1607 (56.6)	1233 (43.4)		
Higher	333 (7.4)	235 (70.6)	98 (29.4)		
**Wealth index**				2.419	0.298
Poor	1522 (34.0)	852 (56.0)	670 (44.0)		
Middle	698 (15.6)	395 (56.6)	303 (43.4)		
Rich	2252 (50.4)	1316 (58.4)	936 (41.6)		
**Religion**				25.763	< 0.001
No religion	245 (5.5)	106 (43.3)	139 (56.7)		
Christians	4183 (93.5)	2426 (58.0)	1757 (42.0)		
Muslim	13 (0.3)	7 (53.8)	6 (46.2)		
Traditional	26 (0.6)	20 (76.9)	6 (23.1)		
Others	5 (0.1)	4 (80.0)	1 (0.1)		
**Smoking**				0.771	0.38
No	4453 (99.6)	2554 (57.4)	1899 (42.6)		
Yes	19 (0.4)	9 (0.4)	10 (0.5)		
**Respondent currently working**				13.203	< 0.001
No	2529 (56.6)	1509 (59.7)	1020 (40.3)		
Yes	1943 (43.4)	1054 (54.2)	889 (45.8)		
**Respondent drinking alcohol**				36.47	< 0.001
No	4005 (89.6)	2356 (58.8)	1649 (41.2)		
Yes	466 (10.4)	206 (44.2)	260 (55.8)		
**Husband/Partner’s educational level**				26.135	< 0.001
No education	59 (1.3)	37 (62.7)	22 (37.3)		
Primary	898 (20.1)	487 (54.2	411 (4.8)		
Secondary	2947 (65.9)	1665 (56.5)	1282 (43.5)		
Higher	2947 (65.9)	345 (67.3)	168 (32.7)		
Don’t know	53 (1.2)	28 (52.8)	25 (47.2)		
**Partner’s occupation**				10.297	0.006
Not working	627 (14.2)	380 (60.6)	247 (39.4)		
Agricultural	539 (12.2)	278 (51.6)	261 (48.4)		
Non Agricultural	3261 (73.7)	1884 (57.8)	1377 (42.2)		
**Women empowerment**				0.177	0.674
No	20 (0.9)	10 (50.0)	10 (50.0)		
Yes	2203 (99.1)	1205 (54.7)	998 (45.3)		
**Father beat Mother**				103.49	< 0.001
No	2968 (66.4)	1860 (62.7)	1108 (37.3)		
Yes	1504 (33.6)	703 (46.7)	801 (53.3)		
**Parity†**				3.762	0.288
None	206 (4.6)	131 (63.6)	75 (36.4)		
1–2	2027 (45.3)	1163 (57.4)	864 (42.6)		
3–4	1721 (38.5)	978 (56.8)	743 (43.2)		
5+	518 (11.6)	291 (56.2)	227 (43.8)		
**Media exposure**				5.877	0.015
No	982 (22.0)	596 (60.7)	386 (39.3)		
Yes	3490 (78.0)	1967 (56.4)	1523 (43.6)		
**Partners drinking alcohol**				150.7	< 0.001
No	2718 (60.8)	1756 (64.6)	962 (35.4)		
Yes	1754 (39.2)	807 (46.0)	947 (54.0)		
**Age difference† (reference)**				2.426	0.489
Women same age or older than man	320 (7.2)	191 (59.7)	129 (40.3)		
Man older by 5 years	2041 (45.7)	1147 (56.2)	894 (43.8)		
Man older by 10 years	1385 (31.0)	799 (57.7)	586 (42.3)		
Man older by more than 10 years	724 (16.2)	425 (58.7)	299 (41.3)		
**Number of Wives†**				2.293	< 0.001
Monogamy	4012 (90.9)	2351 (58.6)	1661 (41.4)		
Polygamy	403 (9.1)	187 (46.4)	216 (53.6)		

†Re-categorized *: column percentage, **: row percentage. p ≤ 0.05; N = number of respondents

Majority, 56.6% (2529) of the women were unemployed; while about three fourth 73.7% (3261) of their partners were employed in non-agricultural works. One third, 33.6% (1504) of the women claimed that their fathers ever beat mothers. Majority, 45.3% (2027) of the women had one or two living children. Almost all, 99.1% (2203) of the respondents were empowered; and more than three fourth, 78.0% (3490) of the respondents were exposed to mass media. A significant number 45.7% (2041) of the respondents were 5 years less than their partners. Lastly, nine out of ten, 90.9% (4012) of the respondents’ partners had no other wives (mostly monogamous relations).

### Key socio-demographic characteristics and association with domestic violence among respondents

Correlations between key socio-demographic characteristics and outcome variables were computed (Table 1). Among these key socio-demographic factors are: age, educational level, employment and alcohol drinking behavior of respondents and their partners, abusive fathers of respondents, media exposure and number of wives were found to be significantly associated with domestic violence (Table 1).

### Trends in domestic violence

**Fig. 1:**
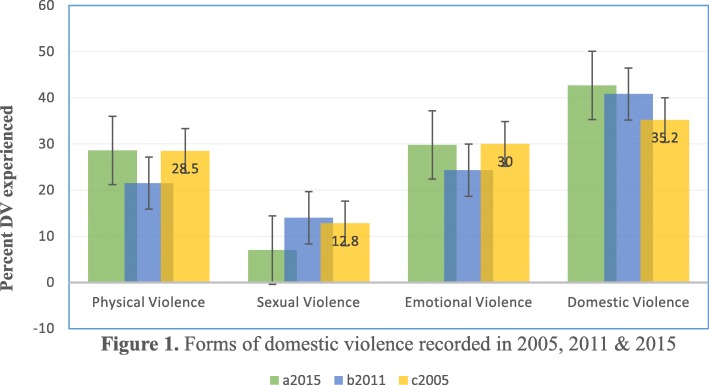
Forms of domestic violence recorded in 2005, 2011 & 2015

**Fig. 2:**
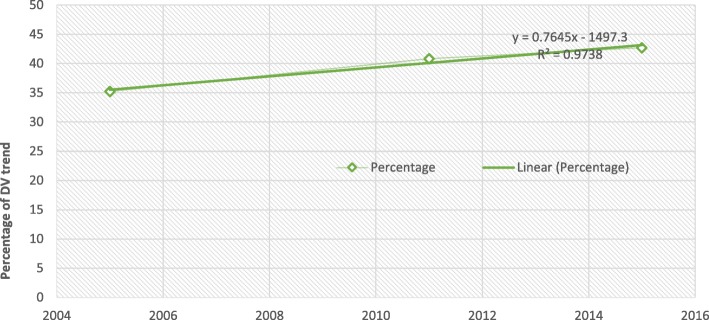
Trends of domestic violence recorded in 2005, 2011 & 2015 among respondents

Trend of various household violence experienced by Zimbabwean women based on nationally representative data for 2005, 2011 and 2015 Zimbabwe Demographic Health Survey were statistically described (Fig. 1). The patterns of domestic violence prevalence for about 10 years was steadily increasing, the percentage of women who experienced domestic violence was 35.2% (1274) in 2005 and increased to 40.8% (1584,) in 2011 and further increased to 42.7% (1909) in 2015 which represent the most kind of violence (Fig. 2), followed by emotional violence, 30% (1086) in 2005 and increased to 24.3% (943) in 2011 and further increased to 29.8% (1333) in 2015 while physical violence was 28.5% (1032) in 2005 and increased to 21.5% (835) in 2011 and further increased to 28.6% (1279) in 2015, sexual violence was the least recorded 12.8% (463) in 2005 and increased to 14% (544) in 2011 and further increased to 7% (313) in 2015 violence among married or co-habitating Zimbabwean women (Fig. 1).

### Factors associated with domestic violence

Domestic violence and its associated factors among the studies population are shown in Table 2 below. After adjusting the confounding variables, respondents aged 40–49 years were 54% (827) less likely to experience domestic violence as compared to those aged 15–19 years (AOR = 0.44; CI (95%) = 0.24–0.83). Respondents currently working were 1.27 times more likely to experience domestic violence as compared to their unemployed counterparts (AOR = 1.27; CI (95%) =1.00–1.61). Similarly, women who drink alcohol were 1.45 times more likely to experience domestic violence compared to their counterparts (AOR = 1.45; CI (95%) =1.09–1.92). Moreover, respondents whose husband had experienced domestic violence (their fathers beating their mothers) were 1.77 times more likely to experience Violence than those women with different family history (AOR = 1.77; CI (95%) =1.47–2.14). Women who had more than 5 children were 1.82 times more likely to experience violence than women without children (AOR = 1.82; CI (95%) =1.03–3.22). Respondents whose partners drink alcohol were 2.12 times more likely to experience violence than their counterparts who do not drink alcohol (AOR = 2.12; CI (95%) =1.76–2.55). Women who were married to a man with more than one wife were 1.94 more probable to experience violence than their monogamous counterparts (AOR = 1.94; CI (95%) = 1.42–2.65).

**Table 2:** Logistic regression model of association between socio-economic characteristics and Domestic Violence among reproductive women aged 15–49 in Zimbabwe, 2015

Variable	Crude			Adjusted		
	OR	CI (95%)	p	AOR	CI (95%)	p
**Age**^**a**^**(Ref = 15–19)**						
20–29	1.01	(0.76–1.35)	0.928	0.79	(0.45–1.39)	0.413
30–39	0.91	(0.68–1.21)	0.506	0.65	(0.36–1.17)	0.151
40–49	0.64	(0.47–0.87)	0.005	0.44	(0.24–0.83)	0.011
**Residence (Ref = Urban)**						
Rural	1	(0.89–1.13)	0.949	0.85	(0.63–1.15)	0.294
**Highest education level (Ref = No Education)**						
Primary	1.08	(0.62–1.88)	0.774	0.87	(0.34–2.23)	0.773
Secondary	1.03	(0.60–1.78)	0.904	0.94	(0.37–2.42)	0.903
Higher	0.56	(0.31–1.01)	0.055	0.66	(0.24–1.79)	0.415
**Wealth index**^**a**^**(Ref = Poorest)**						
Middle	0.98	(0.81–1.17)	0.788	0.99	(0.73–1.33)	0.934
Rich	0.9	(0.79–1.03)	0.134	0.78	(0.55–1.12)	0.176
**Religion**^**a**^**(Ref = No religion)**						
Christians	0.55	(0.43–0.72)	< 0.001	0.67	(0.43–1.05)	0.08
Muslim	0.65	(0.21–2.00)	0.457	0.76	(0.13–4.39)	0.764
Traditional	0.23	(0.09–0.59)	0.002	0.4	(0.10–1.52)	0.177
Others	0.19	(0.02–1.73)	0.141	–	–	–
**Smoking**^**a**^**(Ref = No)**						
Yes	1.49	(0.61–3.68)	3.383	0.94	(0.29–3.04)	0.923
**Respondent currently working (Ref = No)**						
Yes	1.8	(1.49–2.19)	< 0.001	1.27	(1.00–1.61)	0.047
**Respondent drinking alcohol (Ref = No)**						
Yes	1.8	(1.49–2.19)	< 0.001	1.45	(1.09–1.92)	0.001
**Husband/partner’s educational level (Ref = No education)**						
Primary	1.42	(0.82–2.44)	0.207	1.57	(0.52–4.72)	0.421
secondary	1.29	(0.76–2.21)	0.342	1.39	(0.47–4.13)	0.555
Higher	0.82	(0.47–1.43)	0.484	1.07	(0.35–3.30)	0.907
Don’t know	1.5	(0.71–3.19)	0.291	1.4	(0.37–5.34)	0.618
**Partners occupation**^**a**^**(Ref = Not working)**						
Agricultural	1.44	(1.14–1.82)	0.002	0.98	(0.65–1.48)	0.919
Non Agricultural	1.12	(0.94–1.34)	0.188	0.88	(0.62–1.26)	0.5
**Women empowerment**^**a**^**(Ref = No)**						
Yes	0.83	(0.34–2.00)	0.675	0.71	(0.28–1.76)	0.456
**Father beat mother (Ref = No)**						
Yes	1.91	(1.69–2.17)	< 0.001	1.77	(1.47–2.14)	< 0.001
**Parity**^**a**^**(Ref = None)**						
1–2	1.3	(0.96–1.75)	0.086	1.29	(0.80–2.09)	0.295
3–4	1.33	(0.98–1.79)	0.064	1.53	(0.93–2.50)	0.093
5+	1.36	(0.98–1.90)	0.068	1.82	(1.03–3.22)	0.038
**Media exposure**^**a**^**(Ref = No)**						
Yes	1.2	(1.03–1.38)	0.015	1.28	(0.97–1.69)	0.087
**Partners drinking alcohol (Ref = No)**						
Yes	2.14	(1.90–2.42)	< 0.001	2.12	(1.76–2.55)	< 0.001
**Age difference**^**a**^**(Ref = women same age or older than man)**						
Man older by 5 years	1.15	(0.91–1.47)	0.212	1.36	(0.95–1.95)	0.212
Man older by 10 years	1.09	(0.85–1.39)	0.514	1.25	(0.86–1.81)	0.244
Man older by more than 10 years	1.04	(0.80–1.36)	0.765	1.1	(0.73–1.65)	0.642
**Number of other Wives**^**a**^**(Ref = monogamy)**						
Polygamy	1.63	(1.33–2.01)	< 0.001	1.94	(1.42–2.65)	< 0.001

^**a**^***Re-categorized***

*OR refers to Odd Ratio; AOR refers to Adjusted Odd Ratio; CI refers Confidence Interval and p refers to the p-value at p = 0.05*

In addition, religion, partner’s occupation and media exposure were significantly correlated with domestic violence in the crude analysis but the association disappeared after controlling for confounding variables. However, variables like types of residence, respondents and their partner’s educational level, respondents smoking habit, wealth index and age differentials recorded no significant associations with domestic violence; even though, variables perceived to be associated with Domestic Violence were included in the study.

## Discussion

The study explored factors associated with domestic violence among currently married women and a decadal trends of such incidences in Zimbabwe using the DHS data. The trends showed that from 2005 to 2015; there has been sharp increases in domestic violence from 35.2% in 2005 to 40.8% in 2011 and peaked to 42.7% in 2015. The study revealed that old age (40–49) remained a protective factor for Zimbabwean women against acts of domestic violence of all forms. Working women, respondent who drinks alcohol, women whose husbands ever experienced domestic violence by their fathers beating their mothers, women with 5 or more children, women whose partners drink alcohol and those married to men with more than one wife (those in polygamous relations) were more likely to experience acts of domestic violence.

Empirical literature demonstrates that; young women have a better understanding of the criminal nature of domestic violence than elderly women; however, young women are less likely to understand the complexities of domestic violence in relationships such as the range and seriousness of behavior that potentially predispose them to domestic violence compared to elderly women [7]. Thus, previous studies revealed the vulnerability of young women to domestic violence which is consistent with the present study [12, 15]. Our study established that; women aged 40–49 are less likely to experience domestic violence compared to young women aged 15–19 years. There was however, no significant relationship between partner’s age differences and acts of domestic violence.

Women residing in rural settings are usually less likely to access higher education as majority drop out of primary schools and end up marrying without any employable skills. They become unemployed housewives and dependent on their husbands as they are not educated and empowered predisposing them to violence. This is contrary to women who reside in urban areas with wider access to higher education and opportunities; and therefore less likely to experience domestic violence. This is in consonance with several studies affirming that women who reside in rural areas are more likely to experience domestic violence compared to women residing in urban areas [10, 28]. However, our study contradicts these findings above as there is no significant relationship between place of residence and acts of domestic violence. This may be attributable to strong socio-cultural influence in Zimbabwean society (women) and rising levels of literacy among contemporary women.

Women education is one of the most important tools in the world, the amount of education a person receives usually dictates the type of lifestyle she will be able to lead and how much she will earn. Education equips and empowers an individual with knowledge to reason independently for better choices and decision-making in life [29]. Those with more education are deemed to communicate better, and this ability may serve as a protective factor against domestic violence. Earlier studies have found that higher educational level (secondary level and above) for both women and their partners is a proven protective shield against domestic violence [12, 15]. However, other studies have revealed that educational differences between couples is a potential risk factor for domestic violence [25, 30]. Our study; however, showed that wives (women) and husband’s educational level have no significant correlation with acts of domestic violence among married women in Zimbabwe.

Religious people across all denominations are not exempted from domestic abuse; however, there appears to be a serious lack of understanding regarding abuse and the dynamics of abusive relationships and their impact upon the lives of people involved within such religious denominations generally. This study registered no significant relationship between domestic violence and religion. Alcohol use has direct effects on human physico-cognitive function, reducing self-control and leaving individuals less capable of negotiating a non-violent resolution to conflict within relationships [7, 21]. Frequent and excessive drinking by one partner can birth financial and childcare problems, infidelity or other family issues. Results from this study is consistent with earlier studies that; women whose husbands drink alcohol were more likely to experience domestic violence compared to single women. Most previous studies have found a strong association between alcohol use by either women or husbands with domestic violence [7, 31, 32]. For example; a study conducted in Ghana revealed that women whose husbands drink alcohol were 2.5 more likely to experience domestic violence compared to women whose husbands do not drink [7]. Furthermore, it was also revealed that between 25 and 50% even up to 70% of those who perpetrate domestic abuse drink alcohol at the time of assault [21].

Employment makes women independent as they are able to earn their own income. There was no significant relationship among wealth index and respondents’ partners’ occupations. It is reported that women who are employed are less likely to experience domestic violence compared to unemployed women [12, 21]. However, a study conducted in Rwanda [24] showed that women who earn more income than their spouses are more likely to experience violence than those women who earn less or the same as their spouses [24]. Perhaps, the abuse in some cases has to do with an unconscious fear of losing a partner which is more attractive “on the market” due to their socio-economic status. Thus, we report that, working women were more likely to experience domestic violence compared to non-working women which contradicts reports by Mohamadian et al. [30].

Again, men who have been exposed to violence during their childhood are more likely to do violence in their families; it is argued that because they had learned this behavior from their families. Previous study revealed that women with husbands who experienced their father beating their mother are more likely to experience domestic violence than those without past domestic violence experiences [7]. This is in agreement with our findings that women with husbands who have experienced domestic violence (their fathers beating their mothers) are more likely to experience domestic violence than those without such past experiences [7]. We however recorded no significant relationship among parity, women empowerment and mass media exposure among the studied subjects. This affirms a similar study conducted in Ghana which also recorded no significant relationship between parity and acts of domestic violence [7].

Ashimi et al. [33] established that women who were married to a polygamous husband (with two or more wives) were more likely to experience domestic violence compared to those in monogamous families [33]. This is comparable to the current study where women married to a polygamous husband (with more than one wife) were at higher risk of being victims of acts of domestic violence. This is further authenticated by a study conducted in Sudan where women from polygamous families run higher risks of domestic violence compared to women in monogamous families [8].

### Limitation and strength of the study

This is a nationally representative study to explore the extent of domestic violence among married women in Zimbabwe. The study revealed an increasing trend in domestic violence against married Zimbabwean women. The study provides empirical evidences for an urgent need to intensify education on women’s rights and domestic violence by the Zimbabwean government and relevant stakeholders. The study however; was not without limitations. Same sex relationships were not included in the study because it is against the marital laws of Zimbabwe and are not recognized by the cultural and societal norms of Zimbabwe. Cultural acceptance of violence including sexual violence, as a private affair hinders external intervention and prevents those affected from speaking out and gaining support. Future studies may examine these two variables to better understand their effects on domestic violence in Zimbabwe. As a cross-sectional study, it is inevitably oversimplified to draw a causal inference.

## Conclusion

Domestic violence is strongly associated with women whose husbands drink alcohol, those whose husbands have past domestic violence experiences (their fathers beating their mothers) and women of polygamous husbands (had more than one wife). The government of Zimbabwe ought to work closely with organizations that stand against domestic violence to end this public health canker across the country. This will help to improve the welfare of citizens (especially women) and the health of the national economy; thus, costs incurred by police, hospital and health services, legal courts, social support services will be reduced and channeled to other important services. This will eventually breeds healthy families and citizens for full participation in the development of their community and Zimbabwe as a whole. Advocacy groups, the media and civil society organizations should intensify efforts against domestic violence; rights and privileges of women should be upheld across the country.

## Data Availability

The datasets generated and/or analysed during the current study are available in the [Zimbabwe Demographic Health Survey (ZDHS, 2005–2006, 2010–2011 and 2015)] repository, [accessible online http://dhsprogram.com/].

## Abbreviations

CDC: Center for Disease Control and Prevention
DHS: Demographic and Health Surveys
DV: Domestic Violence
ICF: Inner City Fund
IUD: Intra-Uterine Device
MDGs: Millennium Development Goals
SPSS: Statistical Packages for Social Sciences
USAID: United States Agency for International Development
ZDHS: Zimbabwe Demographic and Health Surveys
ZIMSTAT: Zimbabwe National Statistics Agency
